# A droplet digital PCR (ddPCR) assay to detect *Helicoverpa armigera* (Lepidoptera: Noctuidae) in bulk trap samples

**DOI:** 10.1371/journal.pone.0178704

**Published:** 2017-05-31

**Authors:** Frida A. Zink, Luke R. Tembrock, Alicia E. Timm, Roxanne E. Farris, Omaththage P. Perera, Todd M. Gilligan

**Affiliations:** 1 Department of Bioagricultural Sciences and Pest Management, Colorado State University, Fort Collins, Colorado, United States of America; 2 USDA-APHIS-PPQ-Science & Technology, Mission Laboratory, Edinburg, Texas, United States of America; 3 USDA-ARS Southern Insect Management Research Unit, Stoneville, Mississippi, United States of America; 4 USDA-APHIS-PPQ-Science & Technology, Identification Technology Program, Fort Collins, Colorado, United States of America; Natural Resources Canada, CANADA

## Abstract

Moths in the genus *Helicoverpa* are some of the most important agricultural pests in the world. Two species, *H*. *armigera* (Hübner) and *H*. *zea* (Boddie), cause the majority of damage to crops and millions of dollars are spent annually on control of these pests. The recent introduction of *H*. *armigera* into the New World has prompted extensive survey efforts for this species in the United States. Surveys are conducted using bucket traps baited with *H*. *armigera* pheromone, and, because the same pheromone compounds attract both species, these traps often capture large numbers of the native *H*. *zea*. Adult *H*. *armigera* and *H*. *zea* are very similar and can only be separated morphologically by minor differences in the genitalia. Thus, a time consuming genitalic dissection by a trained specialist is necessary to reliably identify either species, and every specimen must be dissected. Several molecular methods are available for differentiating and identifying *H*. *armigera* and *H*. *zea*, including two recently developed rapid protocols using real-time PCR. However, none of the published methods are capable of screening specimens in large batches. Here we detail a droplet digital PCR (ddPCR) assay that is capable of detecting a single *H*. *armigera* in a background of up to 999 *H*. *zea*. The assay has been tested using bulk extractions of 1,000 legs from actual trap samples and is effective even when using poor quality samples. This study provides an efficient, rapid, reproducible, and scalable method for processing *H*. *armigera* survey trap samples in the U.S. and demonstrates the potential for applying ddPCR technology to screen and diagnose invasive species.

## Introduction

Moths in the genus *Helicoverpa* are some of the most important agricultural pests in the world [1, 2, 3]. Two species, *H*. *armigera* (Hübner) and *H*. *zea* (Boddie), cause the majority of damage to crops and millions of dollars are spent annually on control of these pests [2,4]. Prior to 2012, *H*. *zea* and *H*. *armigera* were geographically separated, with *H*. *zea* widespread in both North and South America and in the Caribbean, and *H*. *armigera* prevalent throughout much of the Old World, including Europe, Asia, Africa, and Australasia [1, 2]. *Helicoverpa armigera* was first discovered in the New World in Brazil in 2012/2013, and it has since spread to much of South America and the Caribbean, including Puerto Rico [5, 6, 7, 8]. Although not yet present in the continental U.S., three individuals were captured in Florida in 2015 suggesting that establishment is imminent [9, 10].

The extreme similarity of *H*. *armigera* to the native *H*. *zea* makes detection and identification of *H*. *armigera* difficult. It is hypothesized that *H*. *zea* split from *H*. *armigera* by a founder event from *H*. *armigera* stock colonizing the New World approximately 1.5 million years ago [11,6]. This hypothesis is supported by DNA data as well as mating compatibility between the two species and very similar morphology. Only minor differences in genitalia separate the two species morphologically [12, 13], and dissection by a trained specialist is necessary to reliably identify either species.

The need to dissect every specimen for positive identification is especially problematic when conducting surveys for *H*. *armigera* adults. In the U.S., domestic monitoring for invasive pests is conducted through the USDA’s Cooperative Agricultural Pest Survey (CAPS) program. The CAPS program is designed to “provide a survey profile of exotic plant pests in the United States deemed to be of regulatory significance,” and it includes adult surveys for many lepidopteran pests, including *H*. *armigera*. Surveys for male *H*. *armigera* are usually performed using pheromone-baited wire-cone traps [14] or plastic bucket traps, although plastic bucket traps were economically more efficient [15, 16]. The pheromone lure, a rubber septum impregnated with a mixture of Z11-16:Ald, Z9-16:Ald, and BHT [16], is placed in the bucket trap along with an insecticidal strip. The same pheromone compounds are used by *H*. *armigera* and *H*. *zea* [12], thus traps baited with *H*. *armigera* pheromone also attract male *H*. *zea*, often in large numbers; we have observed bucket traps from CAPS surveys which contain up to 500 *H*. *zea* individuals. In surveys where hundreds of non-targets are encountered per trap, it is simply not practical to use morphology as the primary means of identification.

Many molecular methods have been used to identify and separate *H*. *armigera* from its close relatives. Ming and Wang were able to differentiate between *H*. *armigera* and *H*. *assulta* using AFLP analysis [17], and Kranthi et al. [18] used RFLP analysis for separating the same taxa. Behere et al. [11] also used RFLP analysis to differentiate between *H*. *armigera* and *H*. *zea* with mitochondrial genes. Several studies have used COI sequencing and DNA barcoding to separate *Helicoverpa* species. Li et al. [19] used COI sequences to separate *H*. *armigera* and *H*. *assulta*. Tay et al. [6], Leite et al. [20], Mastrangelo et al. [21] (and others) used COI data to separate and characterize *H*. *armigera* and *H*. *zea* populations in the New World. Nagoshi et al. [22] used COI along with an additional marker, the Z-linked *Tpi* marker, to establish a method of identifying potential *H*. *zea*-*H*. *armigera* hybrids. Two studies have focused on the use of “second generation” real-time PCR to identify *Helicoverpa* species. Gilligan et al. [7] developed an assay based on dual-labeled hydrolysis probes capable of identifying *H*. *armigera* and *H*. *zea* using the Internal Transcribed Spacer 2 (ITS2) gene. Perera et al. [23] used real-time PCR in conjunction with dissociation curve analysis to identify *H*. *armigera* and *H*. *zea* using the Internal Transcribed Spacer 1 (ITS1) gene, and they also demonstrated that *H*. *armigera* DNA could be detected in a background of *H*. *zea* DNA at ratios of 1:24 when specimens of both species were extracted together. With the exception of Perera et al. [23], each of the aforementioned studies focus on identifying individual specimens. None of the studies listed here are capable of rapidly detecting *H*. *armigera* in a single reaction using bulk samples from pheromone traps that may contain up to 500 or more individual moths, predominantly consisting of *H*. *zea*.

Third generation PCR technology, specifically droplet digital PCR (ddPCR), has the potential to fulfill the need for a rapid, reproducible diagnostic method for detection and species determination of individual specimens from bulk samples. This relatively new technology allows for sensitive detection of very small amounts of target DNA by partitioning a single PCR mixture into approximately 20,000 individual nanoliter-sized water-in-oil droplets [24]. After thermal cycling, the droplets are read one-by-one in a specialized machine and assigned a “1” if positive and a “0” if negative (the basis for the term digital). Partitioning the PCR into thousands of individual droplets is functionally equivalent to running a separate PCR on each individual DNA fragment, leading to reliable amplification of target sequences and decreased sensitivity to PCR inhibitors. Furthermore, partitioning allows for, on average, less than one target sequence per droplet permitting the calculation of the absolute amount of target DNA in the total PCR mix [24]. Initially, detection of positive droplets in ddPCR experiments was done using hydrolysis probes, but recent advancements in ddPCR technology have allowed for a streamlined approach to identifying positive droplets with the use of EvaGreen, an intercalating DNA dye [25]. This new system allows for the direct identification of PCR products without the use of probes, reducing per-sample cost [26].

Many ddPCR studies have adapted real-time PCR methods to determine the functionality of ddPCR in previously established assays [27, 28, 29, 30]. For example, ddPCR has been used to observe fecal contamination and the presence of invasive species in environmental DNA samples, in the detection of GMO plant specimens, and to determine the prevalence of rare mutations in circulating, cell-free DNA in patient blood samples [27, 28, 30, 24]. These types of studies set a precedent for our experiments, as they represent varying situations in which small amounts of target DNA are identified within a large background of non-target DNA. Here we present a method for identifying low levels of *H*. *armigera* DNA in a background of *H*. *zea* DNA using EvaGreen ddPCR. The assay is tested using actual trap samples for bulk extractions consisting of legs from up to 1,000 individual specimens.

## Materials and methods

### Sample collection and preparation

Specimens of *H*. *zea* used in this study were obtained from USDA CAPS pheromone traps deployed across the United States between 2012 and 2016. Those specifically used for batch extraction testing were collected using pheromone traps at the Colorado State University College of Agricultural Science Agricultural Research & Education Center (ARDEC) in September, 2016 and obtained from CAPS traps deployed in Oregon in August and September, 2015. Specimens of *H*. *armigera* used in this study were obtained from a captive laboratory colony in Germany (established using individuals from Queensland, Australia) and from specimens collected in South Africa. All specimens were identified to species by morphological dissection following Brambila [13] and/or probe-based real-time PCR following Gilligan et al. [7]. CAPS surveys were conducted on state or private land with permission of the land owner and specimens were obtained under the authority of the United States Department of Agriculture. Field collections of *H*. *armigera* in Gauteng Province, South Africa did not require a collecting permit under the Nature Conservation Ordinance of 1983 or the South African National Environmental Management: Biodiversity Act of 2004. No endangered or protected species were collected for this study. The specimens used for this study are representative of the age and condition of insect material expected to be obtained from pheromone traps. Pinned specimens were stored dry; specimens from pheromone traps were stored in envelopes at −50°C until ready for use. Once removed from specimens, individual legs were stored in 100% ethanol at −20°C.

### Single specimen DNA extraction

Genomic DNA was extracted from individual specimens using a Qiagen DNeasy Blood and Tissue Kit (Qiagen, Valecia, California) from single legs of *H*. *armigera* or *H*. *zea*. Dry legs were crushed in a 1.5 mL microcentrifuge tube and incubated in Buffer ATL plus Proteinase K overnight at 56°C in a GeneMate Digital dry bath (BioExpress, Kaysville, Utah). The rest of the extraction was carried out following manufacturer’s instructions and eluted in 100 μL buffer AE. DNA concentration was estimated using a NanoDrop 2000 spectrophotometer (Thermo Scientific, Waltham, Massachusetts), and is reported as the average of two readings per sample.

### DNA batch extraction

DNA was extracted from *H*. *armigera* and *H*. *zea* legs using “squish buffer” as described by Perera et al. [23], modified from Gloor et al. [31]. Briefly, legs from *H*. *armigera* and *H*. *zea* were placed in 1.5 mL microcentrifuge or 7 mL Falcon tubes with a specific number of 2.3 mm zirconia/silica beads (BioSpec Products, Bartlesville, Oklahoma) and ground dry in a Mini-Beadbeater (BioSpec Products) for 55 seconds on high speed. Two beads were used for smaller amounts of tissue, with up to 30 beads for the largest samples. After the legs were reduced to a fine powder, 15 μL/leg of squish buffer (10 mM Tris-HCl, 0.5 mM EDTA, and 12.5 mM NaCl) was added to each tube (large samples were transferred to 50 mL Falcon tubes for extraction). The tubes were incubated at 80°C in an Isotemp 102S water bath (Fisher Scientific, Waltham, Massachusetts) for 2 hours with occasional agitation for large volumes and a shaking table-top Thermomixer FP dry bath (Eppendorf AG, Hamburg, Germany) at 80°C for 2 hours for smaller volumes. Following incubation, large samples were transferred to 1.5 mL microcentrifuge tubes and spun down at 14,000 rpm in a Centrifuge 5418 (Eppendorf AG) for 10 minutes to pellet debris. Small volume samples were transferred to or remained in 1.5 mL microcentrifuge tubes and spun down at 14,000 rpm in an Centrifuge 5418 (Eppendorf AG) for 10 minutes to pellet debris. Supernatant was transferred to clean 1.5 mL microcentrifuge tubes and stored at −20°C until use. Batch leg extractions were carried out in ratios of (*armigera*:*zea*) of: 1:4, 1:9, 1:24, 1:49, 1:99, 1:199, 1:499, and 1:999. Additionally, a sample of 5 legs of *H*. *armigera*, 5 legs of *H*. *zea*, and a No Tissue Control (NTC) were processed in the same manner.

### Real-time PCR and dissociation curve analysis

Dissociation curve differentiation of *H*. *armigera* from *H*. *zea* was carried out as described in Perera et al. [23] based on the Internal Transcribed Spacer 1 (ITS1) region of ribosomal RNA (rRNA). The conserved 18S rRNA region was used as a control. The PCR master mix included the following (primers are listed in Table 1): 0.4 μM 3374Ha_ITS1R, 0.4 μM 3373Ha_Hz_ITS1F, 0.2 μM 3377Hz_ITS1R1, 1.2 μM 3695_18S_1150F, 1.2 μM 3696_18S_1232R, 10 μL Bio-Rad SYBR Taq, and 1.2 μL sterile H_2_O; 2 μL of template DNA (from batch extractions described above) was added for a total reaction volume of 20 μL. Reactions were carried out in white Bio-Rad 96-well plates (Bio-Rad Laboratories Inc., Hercules, California) which were sealed with an optically clear Microseal ‘B’ seal (Bio-Rad Laboratories Inc.). Primers were ordered from Integrated DNA Technologies (IDT, Coralville, Iowa). The plate was placed in a Bio-Rad CFX96 (Bio-Rad Laboratories Inc.) and run on the following cycle: 95°C for 1 min, 95°C for 5 sec, 58.8°C for 30 sec followed by a plate read. Steps 2 and 3 were repeated 39 times after which the melt curve was run from 60.0°C to 95.0°C at 0.1°C per sec with a plate read each second. The resulting peaks were plotted individually using Bio-Rad CFX Manager 3.1 (Bio-Rad Laboratories Inc.).

**Table 1:** Primers used in this study (Tm = melting temperature in °C).

Name	Description	Sequence	Tm	Source
3373Ha_Hz_ITS1-F	Common ITS1 forward primer	5’-GAGGAAGTAAAAGTCGTAACAAGGTTTCC	57.5	Perera et al. 2015
3374Ha_ITS1-R	*H. armigera* ITS1 reverse primer	5’-CGTTCGACTCTGTGTCCTCTAGTGG	60.2	Perera et al. 2015
3377Hz_ITS1-R	*H. zea* ITS1 reverse primer	5’-TTGATTGTTAACGAACGCGCCG	58.7	Perera et al. 2015
3695_18S_1150F	18S rRNA forward primer	5’-GCAGCTTCCGGGAAACCAAA	58.6	Perera et al. 2015
3696_18S_1232R	18S rRNA reverse primer	5’-GCCCTTCCGTCAATTCCTTTAAGT	57.4	Perera et al. 2015

### Droplet digital PCR analysis

Droplet digital PCR (ddPCR) analysis was performed using EvaGreen intercalating DNA dye to detect positive droplets. Before ddPCR, purified DNA samples were digested with HindIII in a reaction containing 5.0 μL 10x HF Buffer (Fisher Scientific, Waltham, Massachusetts), 5.0 μL HF HindIII (Fisher Scientific), 30 μL of sterile H_2_O, and 10 μL DNA at 37°C for 1 hour in a C1000 Touch Thermal Cycler (Bio-Rad Laboratories Inc.) to allow for efficient incorporation of DNA into droplets. After initial testing and optimization, each ddPCR reaction contained 10 μl 2x EvaGreen Supermix (Bio-Rad Laboratories Inc.), 150 nM 3373Ha_Hz_ITS1F forward primer (Table 1), 150 nM 3374Ha_ITS1R reverse primer (Table 1), and sterile H_2_O to bring the per-reaction volume to 18 μL. The master mix was vortexed twice for 10 seconds, and spun down briefly each time. The master mix was aliquoted into 0.2 mL tubes and 2.0 μL of digested DNA was added to each reaction for a final volume of 20 μL.

The PCR mixture (20 μL total) was then added to the middle wells of disposable DG8 Cartridges for the QX100/QX200 Droplet Generator (Bio-Rad Laboratories Inc.), after which 70 μL of EvaGreen droplet generation oil (Bio-Rad Laboratories Inc.) was added to the bottom wells of the same cartridges. The cartridge was sealed with a disposable rubber DG8 Gasket (Bio-Rad Laboratories Inc.) and the cartridge was placed in the Bio-Rad QX200 Droplet Generator System (Bio-Rad Laboratories Inc.) for droplet generation. The final volume of droplets in oil was approximately 40 μL.

After droplet generation, the droplets were transferred from the top well of the DG8 cartridge to an Eppendorf semi-skirted 96-well plate (Eppendorf AG) using an Eppendorf Xplorer Plus 5–100 μL electronic pipettor (Eppendorf AG) set to the lowest draw and expel speed in an excess volume to maximize droplet recovery after transfer. The plate was sealed with a Bio-Rad Pierceable Foil Heat Seal (Bio-Rad Laboratories Inc.) in a PX1 PCR Plate Sealer (Bio-Rad Laboratories Inc.) and sealed for 5 seconds at 180°C. The PCR reaction was carried out in a Bio-Rad C1000 Touch Thermal Cycler (Bio-Rad Laboratories Inc.) with a 96-deep well reaction module using the following program: (1) 95°C for 5 minutes, (2) 95°C for 30 seconds, (3) 57°C for 1 minute, (4) steps 2 and 3 repeated for 39 cycles, (5) 4°C for 5 minutes, (6) 95°C for 5 minutes, and (7) an infinite hold at 4°C. In between each step of the protocol, the ramp rate was 2°C/second to ensure the droplet temperature changes in conjunction with the surrounding oil. Steps 5 and 6 as well as the low infinite hold temperature are specific to EvaGreen ddPCR and are required to cement the spherical shape of the droplets so they can be easily read.

After thermal cycling, the plate was placed in the block of a Bio-Rad QX200 Droplet Reader (Bio-Rad Laboratories Inc.). Droplets were read at a rate of 32 wells/hour and data were analyzed in QuantaSoft version 1.7.4 (Bio-Rad Laboratories Inc.). Single well thresholding was used to group droplets using the software’s default internal algorithm. Poisson statistics were also determined by the software. Error bars represent the Poisson 95% confidence maximum and minimum based on the software’s algorithm. Poisson-corrected copies/droplet (CPD) was calculated using the following equation (Bio-Rad, unpublished):

$$CPD=-ln(1-\frac{positive\;droplets}{total\;droplets})$$

To provide a better estimate of the number of positive and negative droplets and to increase reproducibility of results, the Javascript program “definetherain” [32] (http://www.definetherain.org.uk) was used to set the threshold fluorescence amplitude following each experiment. Positive controls used to calculate fluorescence thresholds consisted of runs using only *H*. *armigera* DNA. All ddPCR data reported are based on thresholds calculated by “definetherain.” In each figure, the amplitude of the droplets is shown in A), positive droplets are blue while negative droplets are grey; the concentrations for each reaction are plotted in B), error bars represent the minimum and maximum Poisson 95% confidence intervals; and the table in C) shows full results from each ddPCR run. The raw data used to produce each figure is provided in S1–S5 Tables.

### Initial ddPCR testing

Initial ddPCR tests were performed with purified undiluted *H*. *armigera* DNA template. Fourteen samples (plus a NTC) were selected with a range of DNA concentrations from 5.35 ng/μL to 878.5 ng/μL, as estimated by NanoDrop. Droplet digital PCR analysis followed the protocol listed above, including DNA digestion.

Initial mixed template testing was performed using DNA from two samples of *H*. *armigera* (HELICOV-121 and 146) and two samples of *H*. *zea* (HELICOV-161 and 171); DNA template concentrations ranged from 171 ng/μL to 411 ng/μL. To ensure that the number of copies of ITS1 did not exceed 100,000, which is the recommended upper limit for absolute quantification with the QX200 [33, 24], DNA template was diluted 100x (based on initial DNA concentration in ng/μL). Diluted DNA template was then mixed in ratios (*armigera*:*zea*) of 1:1, 1:5, and 1:10. Diluted *H*. *armigera* DNA was used as a positive control. This experiment was repeated twice following the ddPCR protocol listed above including DNA digestion.

### Bulk extraction ddPCR testing

The ddPCR protocol outlined above was performed on digested DNA from batch leg extractions in ratios (*armigera*:*zea*) of: 1:4, 1:9, 1:24, 1:49, 1:99, 1:199, 1:499, and 1:999. Each assay included DNA from *H*. *armigera* and *H*. *zea* extracted in the same manner as positive and negative controls, and a NTC. This experiment was performed twice with the same samples. Additional assays were performed with a separate set of samples for high ratios (1:199 to 1:999) and on samples with different DNA concentrations (detailed in the results).

### Limit of detection

The false positive detection rate (FPR) for the assay was estimated in order to calculate the limit of detection (LoD), or the lowest number of positive droplets at which detection of *H*. *armigera* ITS1 is possible. The FPR was estimated by replicating the batch extraction protocol (including DNA digestion) on the following samples: three *H*. *armigera*; three NTCs; two batch extractions of 99 *H*. *zea* individuals; and 32 individually extracted *H*. *zea*. The FPR was calculated by tabulating the total number of positive droplets for *H*. *zea* and dividing by the total number of wells containing *H*. *zea* (34 total). The FPR was used to determine the call threshold, which was then used to determine the LoD. Threshold and LoD values were obtained from lookup tables provided by Bio-Rad based on calculations by Armbruster and Pry [34].

## Results

### *H*. *armigera* was detected in batch extractions using real-time PCR

**Fig 1:**
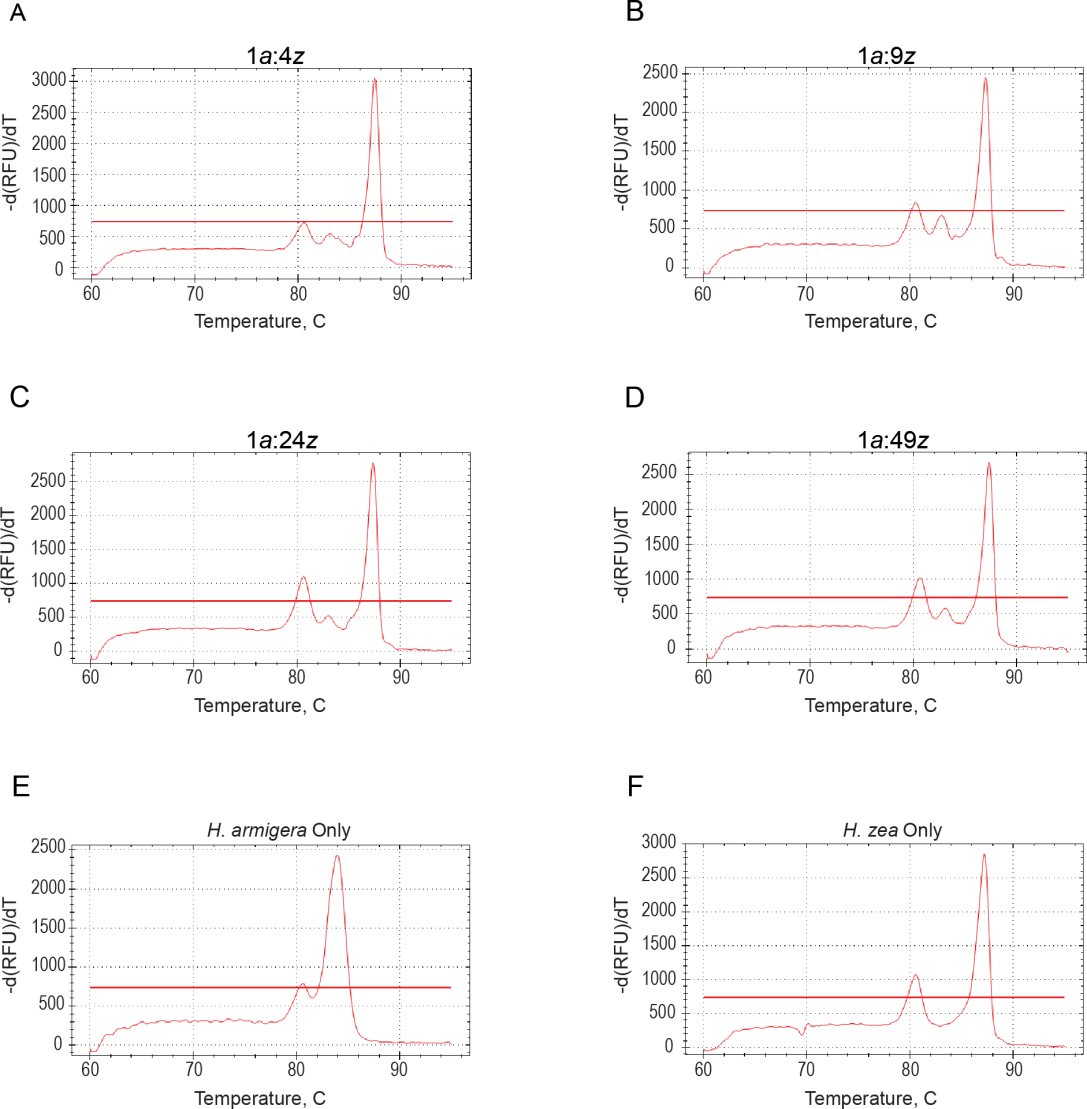
Dissociation melt curves detect *H*. *armigera* in batch extractions at low ratios. Dissociation curve analysis was run to detect a single leg of *H*. *armigera* extracted with increasing numbers of *H*. *zea* legs. ITS1 of *H*. *armigera* was detected in ratios A) 1:4, B) 1:9, C) 1:24, and D) 1:49. The melt curve for *H*. *armigera*-only is shown in E), while the melt curve for *H*. *zea*-only is shown in F). All reactions also include 18S control primers. From left to right, the peaks in each curve represent the melting temperatures of 18S rRNA, *H*. *armigera* ITS1, and *H*. *zea* ITS1 PCR products. The horizontal red line in each graph represents the baseline threshold which is automatically set by the software. Identification of melt peaks is based on the presence of a peak at the expected temperature independent of the baseline threshold.

Perera et al. [23] used pooled *H*. *armigera* and *H*. *zea* legs to demonstrate that *H*. *armigera* could be detected in a background of *H*. *zea* at ratios (*armigera*:*zea*) of 1:19 and 1:24. We replicated this experiment by extracting DNA from combinations of *H*. *armigera* and *H*. *zea* legs in ratios (*armigera*:*zea*) of: 1:4, 1:9, 1:24, 1:49, 1:99, 1:199, 1:499, and 1:999. Additionally, *H*. *armigera*- and *H*. *zea*-only positive and negative controls, as well as NTCs, were run in parallel. *Helicoverpa armigera* was detected using a dissociation melt curve in ratios from 1:4 to 1:49 (Fig 1A–1D). In ratios of 1:99 to 1:999, no *H*. *armigera* sequences were detected, and the results were similar to those in Fig 1F. Peak height for the 18S control is similar to that observed by Perera et al. [23] for batch extracted legs of both species, and is directly related to amplicon length and nucleotide composition. The relatively small peak for ITS1 in *H*. *armigera* is likely due inefficient amplification of this gene region in the presence of competing reverse primer for *H*. *zea* in the multiplex PCR, as discussed by Perera et al. [23].

### *H*. *armigera* DNA was detected in an excess of *H*. *zea* DNA by ddPCR

**Fig 2:**
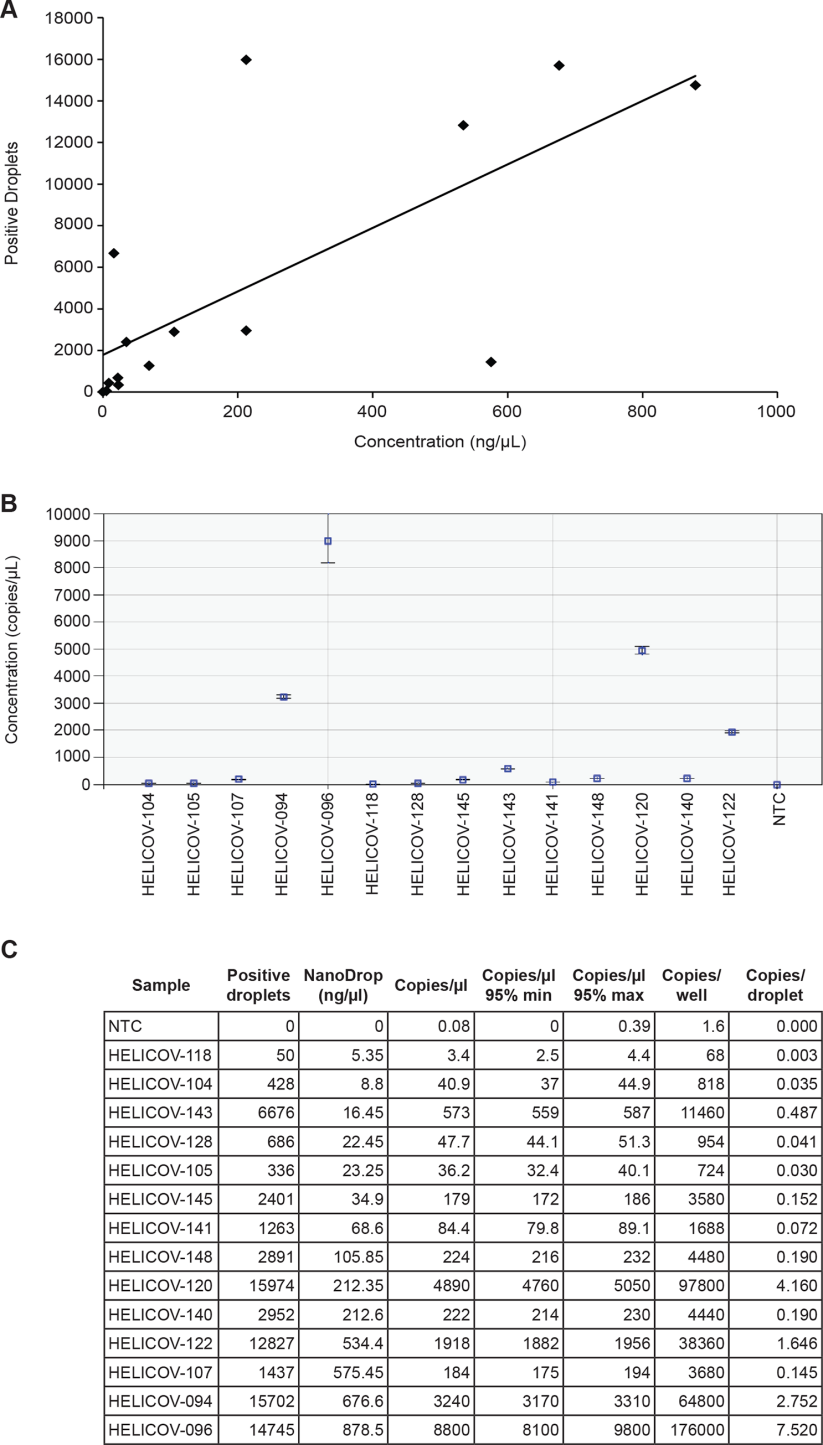
*H*. *armigera* DNA was detected in ddPCR at all concentrations. DNA extractions from 14 *H*. *armigera* specimens were used to run initial tests of ddPCR. *Helicoverpa armigera* was detected in each reaction throughout the range of DNA concentrations (as measured by NanoDrop). The relationship between DNA concentration and positive droplet count is shown in (A). Concentration (copies/μL) is shown in (B); error bars represent the minimum and maximum Poisson 95% confidence intervals for copies/μL. The table in (C) shows NanoDrop readings for each sample as well as the data generated by QuantaSoft and the Poisson corrected copies/droplet.

Initial ddPCR experiments consisted of running *H*. *armigera* DNA extractions in a range of known concentrations to observe the relationship between ng/μL and copies/μL (Fig 2). DNA concentrations of the samples ranged from 5.35 ng/μL to 878.5 ng/μL as measured by NanoDrop. The results showed a rough correlation between DNA concentration and copies/μL (positive droplets; Fig 2) for some samples; however, several samples had similar DNA concentrations but very different numbers of positive droplets (e.g., HELICOV-120 vs. 140).

**Fig 3:**
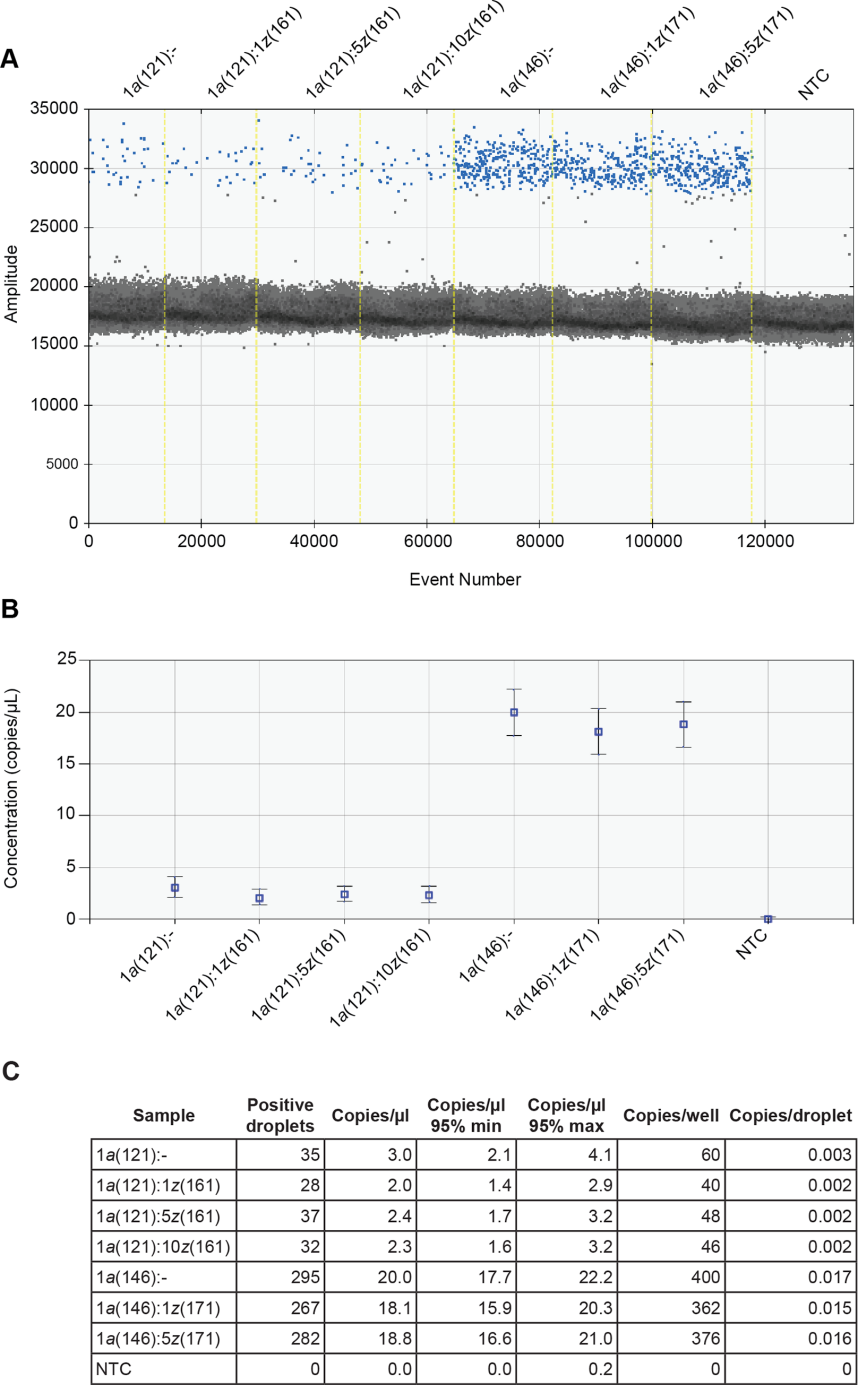
*H*. *armigera* DNA diluted into *H*. *zea* DNA is detectable by ddPCR. Initial mixed testing involved diluting DNA extracted from two specimens of *H*. *armigera* (HELICOV-121 and 146) into DNA extracted from two specimens of *H*. *zea* (HELICOV-161 and 171). *Helicoverpa armigera* ITS1 DNA was detected in each case. The amplitude of recorded droplets is shown in (A); positive droplets are blue, negative droplets are grey. The concentrations of each reaction are plotted in (B); error bars represent the minimum and maximum Poisson 95% confidence intervals for copies/μL. The table in (C) shows the Poisson corrected copies/droplet and the information needed to calculate these values, plus the values for concentration and copies/well.

In order to determine the utility of ddPCR for identification of *H*. *armigera* DNA mixed with *H*. *zea* DNA, we replicated the Perera et al. [23] experiment using the parameters they developed for real-time PCR, in a ddPCR experiment. Diluted DNA template from *H*. *armigera* and *H*. *zea* was mixed in ratios (*armigera*:*zea*) of 1:1, 1:5, and 1:10. Copies of *H*. *armigera* were detected in the positive control, as well as in each of the other mixed template reactions (Fig 3). DNA concentration of *H*. *armigera* did not correlate with the number of copies of ITS1 detected, although copies/μL and positive droplets/well were consistent for each specimen, regardless of the dilution factor (Fig 3A). Sample HELICOV-121 (411 ng/μL average DNA concentration) produced an average of 2.4 copies/μL and sample HELICOV-146 (198 ng/μL average DNA concentration) produced an average of 16.3 copies/μL. Results from this experiment were used to optimize primer concentrations and PCR conditions for all following experiments.

### *H*. *armigera* was detected in every ratio of batch extractions using ddPCR

**Fig 4:**
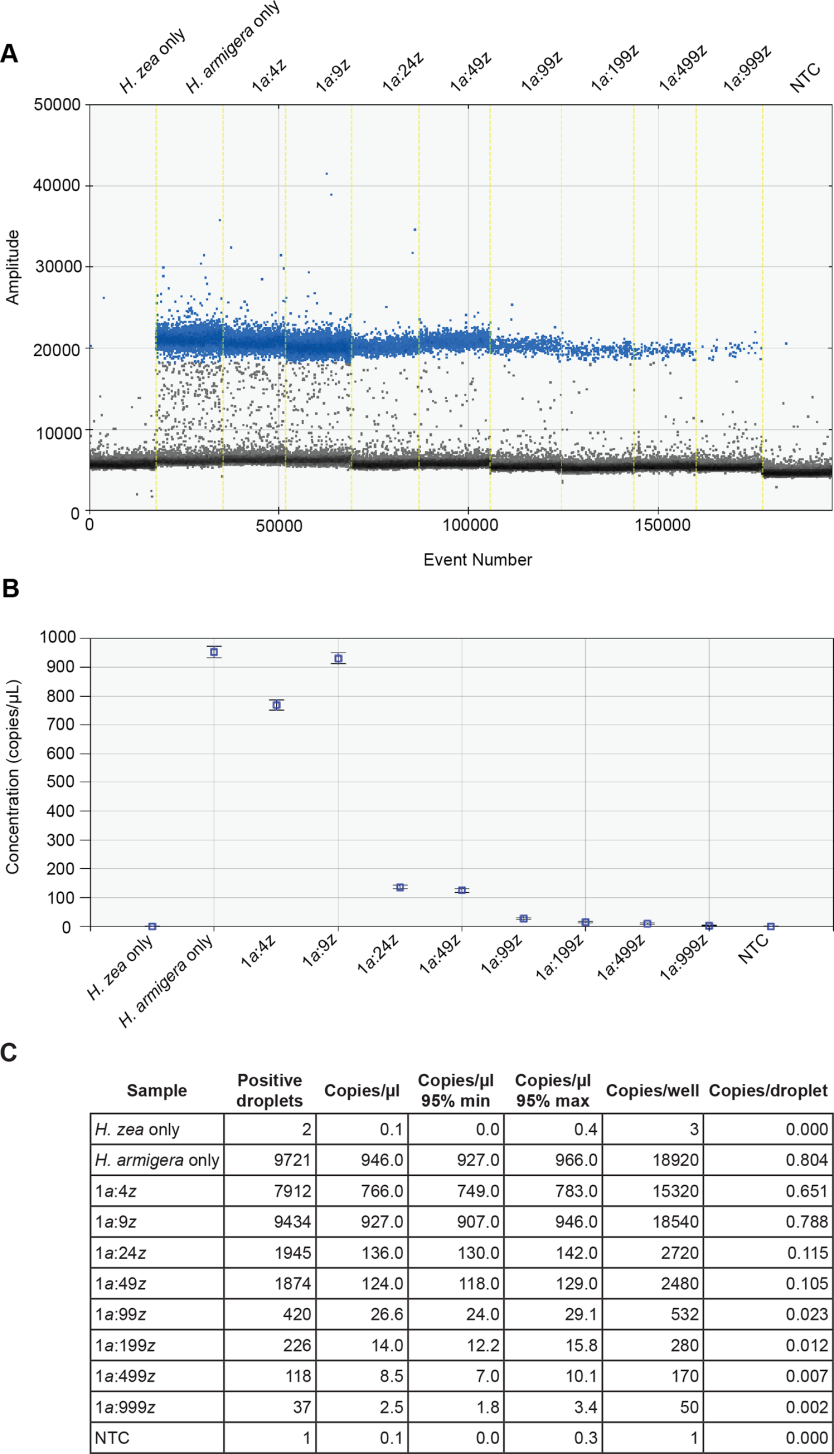
*H*. *armigera* was detected in all batch extractions using ddPCR. DNA was extracted from one leg of *H*. *armigera* pooled with an increasing number of *H*. *zea* legs in ratios (*armigera*:*zea*) of 1:4, 1:9, 1:24, 1:49, 1:99, 1:199, 1:499, and 1:999, and the resulting DNA was tested in the ddPCR assay. *Helicoverpa armigera* ITS1 sequences were detected in all ratios tested, and the results were consistent on a second run of the same samples. See Fig 3 legend for descriptions of A, B, and C.

The same samples used for the real-time PCR dissociation curve analysis were used to test the effectiveness of ddPCR in detecting *H*. *armigera*. The ddPCR assay was tested on batch extractions of *H*. *armigera* and *H*. *zea* legs in ratios (*armigera*:*zea*) of: 1:4, 1:9, 1:24, 1:49, 1:99, 1:199, 1:499, and 1:999. *Helicoverpa armigera* ITS1 sequences were detected in all ratios tested (Fig 4), and the results were consistent on a second run of the same samples. The number of positive droplets/well ranged from 9,721–9,788 for *H*. *armigera*-only to 37–40 for a 1:999 ratio of *armigera*:*zea* (Fig 4; second run not shown).

**Fig 5:**
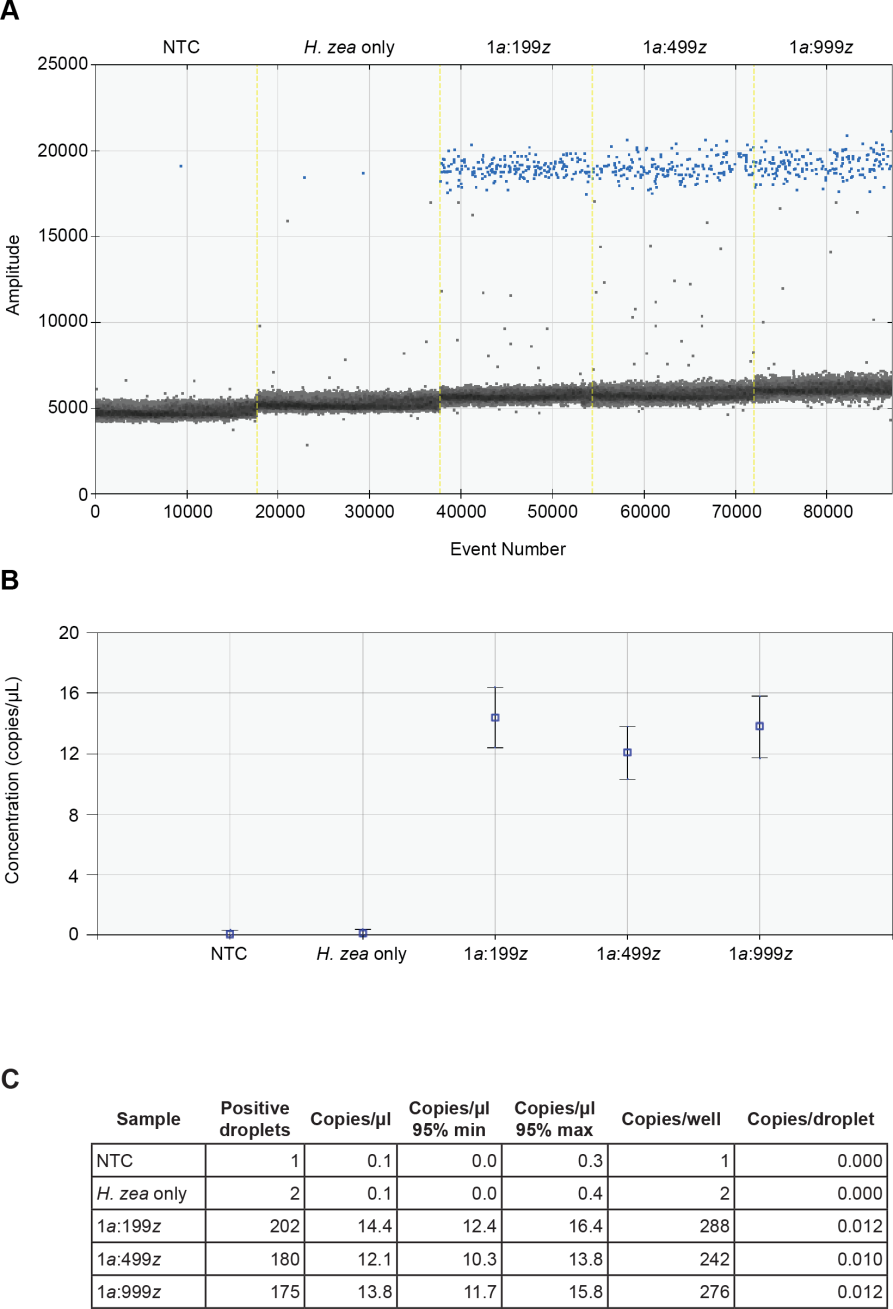
ddPCR results for high ratios of *H*. *zea* to *H*. *armigera* are replicable independent of DNA quality. High ratio tests (1:999, 1:499, and 1:999) were repeated with different samples of *H*. *armigera* having relatively low DNA concentrations (approximately 5 ng/μL). *Helicoverpa armigera* ITS1 sequences were detected in all ratios tested, and the results were consistent on a second run of the same samples. See Fig 3 legend for descriptions of A, B, and C.

Initial bulk extractions used *H*. *armigera* legs from specimens originating from the German lab-maintained colony. To quantify the amount of DNA in these specimens, a single leg from each was retained and DNA was extracted individually using squish buffer. These legs yielded a relatively high concentration of DNA, approximately 300 ng/μL as measured by NanoDrop. To test the effectiveness of the assay with lower DNA concentrations, we used legs from South African *H*. *armigera* museum specimens for which DNA had been previously extracted (using a Qiagen DNeasy Blood and Tissue kit) and quantified (using NanoDrop), with the assumption that all legs from the same specimen will yield a similar amount of DNA. We selected legs from specimens with relatively low DNA concentrations (approximately 5 ng/μL), and repeated high (1:199, 1:499, and 1:999 *armigera*:*zea*) leg ratio extractions and ddPCR. *Helicoverpa armigera* ITS1 sequences were detected in all ratios tested, and the results were consistent on a second run of the same samples (Fig 5; second run not shown). Surprisingly, copies/μL were higher than in the previous experiment (e.g., 14.3 vs. 4.4 for 1:999), suggesting that DNA concentration estimates can be inaccurate for squish buffer extractions and that the yield of bulk extractions likely varies for each sample.

### Limit of detection

**Fig 6:**
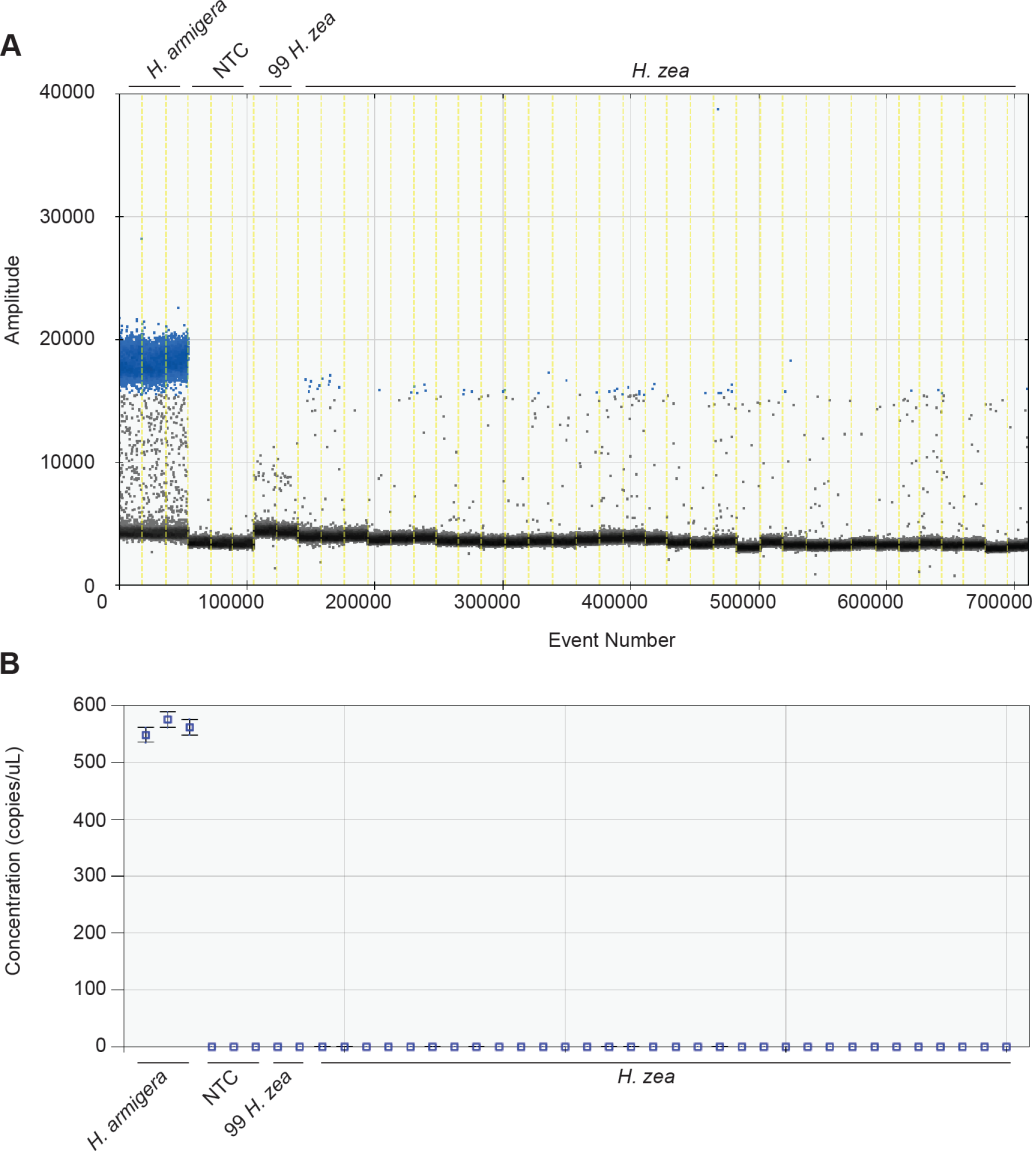
False positive testing results in a LoD of 14 positive droplets/well. False positive rates were estimated with runs of only *H*. *zea*, either singly or bulk extracted. The total number of false positive droplets for all wells (49) was divided by the total number of wells containing *H*. *zea* (34) to obtain a FPR of 1.44 droplets/well. Based on Bio-Rad lookup tables, the threshold to call a well positive at this FPR is 0.32%, which results in a LoD of 14 droplets/well at the 99% confidence level. See Fig 3 legend for descriptions of A, B, and C.

False positive rates were estimated with runs of only *H*. *zea*, either singly or bulk extracted (Fig 6). The total number of false positive droplets for all wells (49) was divided by the total number of wells containing *H*. *zea* (34) to obtain a FPR of 1.44 droplets/well (data in Table 2). Based on Bio-Rad lookup tables, the threshold to call a well positive at this FPR is 0.32%, which results in a LoD of 14 droplets/well at the 99% confidence level. Thus, the assay is able to positively detect *H*. *armigera* ITS1 locus using as few as 14 positive droplets/well, which is approximately half of the lowest number of positive droplets/well obtained from 1:999 *armigera*:*zea* batch extractions (37 droplets/well).

**Table 2:** Data used to calculate the limit of detection (LoD).

Sample	Positive droplets	Copies/μl	Copies/μl 95% min	Copies/μl 95% max	Copies/well	Copies/droplet
*H*. *armigera*	6989	548.00	535.00	561.00	10960.0	0.4660
*H*. *armigera*	7007	575.00	561.00	588.00	11500.0	0.4890
*H*. *armigera*	6651	561.00	548.00	575.00	11220.0	0.4770
NTC	0	0.00	0.00	0.20	0.0	0.0002
NTC	0	0.00	0.00	0.20	0.0	0.0001
NTC	0	0.00	0.00	0.21	0.0	0.0001
*99 H*. *zea*	0	0.00	0.00	0.20	0.0	0.0001
*99 H*. *zea*	0	0.00	0.00	0.21	0.0	0.0001
*H*. *zea*	6	0.40	0.16	0.81	8.0	0.0003
*H*. *zea*	5	0.32	0.11	0.70	6.4	0.0003
*H*. *zea*	0	0.00	0.00	0.19	0.0	0.0000
*H*. *zea*	1	0.07	0.00	0.31	1.4	0.0001
*H*. *zea*	1	0.07	0.00	0.31	1.4	0.0001
*H*. *zea*	4	0.27	0.08	0.63	5.4	0.0002
*H*. *zea*	0	0.00	0.00	0.21	0.0	0.0000
*H*. *zea*	3	0.19	0.05	0.52	3.8	0.0002
*H*. *zea*	2	0.13	0.02	0.41	2.6	0.0001
*H*. *zea*	0	0.00	0.00	0.19	0.0	0.0000
*H*. *zea*	2	0.13	0.02	0.40	2.6	0.0001
*H*. *zea*	1	0.06	0.00	0.31	1.2	0.0001
*H*. *zea*	1	0.06	0.00	0.31	1.2	0.0001
*H*. *zea*	4	0.25	0.08	0.60	5.0	0.0002
*H*. *zea*	4	0.27	0.08	0.63	5.4	0.0002
*H*. *zea*	2	0.14	0.02	0.45	2.8	0.0001
*H*. *zea*	0	0.00	0.00	0.19	0.0	0.0000
*H*. *zea*	1	0.07	0.00	0.31	1.4	0.0001
*H*. *zea*	6	0.39	0.15	0.79	7.8	0.0003
*H*. *zea*	0	0.00	0.00	0.19	0.0	0.0000
*H*. *zea*	0	0.00	0.00	0.20	0.0	0.0000
*H*. *zea*	2	0.13	0.02	0.41	2.6	0.0001
*H*. *zea*	0	0.00	0.00	0.19	0.0	0.0000
*H*. *zea*	0	0.00	0.00	0.20	0.0	0.0000
*H*. *zea*	0	0.00	0.00	0.19	0.0	0.0000
*H*. *zea*	0	0.00	0.00	0.20	0.0	0.0000
*H*. *zea*	1	0.07	0.00	0.34	1.4	0.0001
*H*. *zea*	2	0.14	0.02	0.43	2.8	0.0001
*H*. *zea*	0	0.00	0.00	0.21	0.0	0.0000
*H*. *zea*	0	0.00	0.00	0.20	0.0	0.0000
*H*. *zea*	0	0.00	0.00	0.20	0.0	0.0000
*H*. *zea*	1	0.07	0.00	0.35	1.4	0.0001

## Discussion

Invasive pests present a serious problem with regards to crop losses and decreasing biodiversity. In the United States, *H*. *armigera* presents a serious threat to a number of economically important crops if a population becomes established. Early detection through surveys is an important step in delaying or preventing the incursion of this species. A large number of moths are captured each year in the U.S. during domestic surveys and this number increases as more and more states implement or expand surveys. Because the majority of trap catches are *H*. *zea*, and *H*. *armigera* and *H*. *zea* are externally indistinguishable, a rapid, scalable, reproducible assay to process an entire trap’s contents is required. Here we detail a droplet digital PCR assay that is capable of detecting small amounts of *H*. *armigera* DNA in a background of *H*. *zea* DNA up to ratios (*armigera*:*zea*) of 1:999. The assay has been tested using bulk extractions from actual trap samples, and is effective even when using poor quality samples. The assay has a false positive rate of 1.44 droplets/well and a limit of detection of 14 droplets/well. Thus, the assays can determine, with 99% confidence, that a well contains *H*. *armigera* ITS1 DNA with as few as 14 positive droplets/well.

Our experiments represent a new, practical application for ddPCR that has been tested through all phases of sample processing (collection, extraction, assaying, and data analysis). Most previous studies have used ddPCR to amplify low concentrations of target sequences, such as when detecting endangered species or fecal contamination in water samples, but many rely upon artificially spiking predetermined amounts of purified target DNA into reactions to determine the utility of ddPCR and compare it to real-time PCR (e.g., [27, 28, 24]). This assay is one of the first ddPCR applications capable of detecting rare sequences in moth samples collected from field traps, and not simply in laboratory simulations.

The ddPCR assay described here does have limitations, mainly that it is not capable of identifying the total number of copies of *H*. *armigera* ITS1, and thus providing an estimate of the total number of moths. Variability in sample quality, associated differences in DNA concentration, and multiple genomic copies of ITS1 contribute to this issue. Although there is a general trend that lower DNA concentrations (ng/μL) result in fewer positive droplets, and higher DNA concentrations result in more positive droplets, the relationship is neither consistent nor linear (Fig 2). Consequently, DNA concentration cannot be reliably estimated using positive droplet count. In addition, there is an upper limit to the number of gene copies that can be identified by ddPCR due to the partitioning that occurs. The maximum number of copies/well that can be reliably quantified with ddPCR on the QX200 is 100,000, which is approximately five copies/droplet [33, 24]. Other studies have been able to push that limit, with up to 131,000 copies per well quantified by Pinheiro et al. [35], but absolute quantification becomes less reliable at these upper bounds. Overall copy number quantification, however, is not a requirement for positive detection of *H*. *armigera* ITS1 DNA in a trap sample, thus there is no upper limit for the number of individual *H*. *armigera* that can be included in a single run of the assay.

We were concerned initially at how the quality of DNA from field-collected specimens would influence our ability to identify *H*. *armigera* in bulk extractions. Traps are routinely left in the field for 2–4 weeks, subjecting the specimens to non-ideal conditions (heat, moisture, etc.) that can quickly degrade DNA. Using a large data set of field collected *Helicoverpa* from a previous study [7], we were able to determine that the average minimum DNA concentration for an extraction from a field-collected adult was approximately 5 ng/μL. We tested the assay using specimens of *H*. *armigera* with a similar low DNA concentration and were able to detect *H*. *armigera* ITS1 sequence in ratios (*armigera*:*zea*) as high as 1:999 (Fig 5). These data show that the ddPCR assay is effective for detection across the range of sample quality expected from field-collected specimens.

The number of copies of *H*. *armigera* ITS1 detected in batch extractions varied between ratios, but was relatively consistent for extractions at the same ratios, even when using specimens with different DNA concentrations (e.g., Fig 4 vs. Fig 5). The dilution of *H*. *armigera* DNA into a background of *H*. *zea* DNA resulted in an overall decrease in the number of positive droplets, but the decrease was not linear, likely due to the increased amount of tissue and the corresponding increased amount of extraction buffer used for higher ratios. This decrease in detectable *H*. *armigera* DNA in relation to increasing *H*. *zea* DNA signifies a practical limit to the number of moths that can be processed in a single reaction, although this limit is likely well above 1,000. We were unable to test at higher ratios (5,000 or 10,000) due to the sheer number of specimens required to replicate such an experiment. Practically, the maximum number of *Helicoverpa* that we have observed from a single CAPS pheromone trap is less than 500 individuals, with 50–100 the likely average.

Two factors allow us to be certain that positive droplets detected in the bulk extraction experiments do, in fact, contain copies of *H*. *armigera* ITS1: (1) the amplitude of the positive droplets in comparison to negative droplets and intermediate “rain” is consistent, and (2) positive droplets/well for all experiments exceed the LoD of 14 droplets/well at the 99% confidence level. In cases where the *H*. *zea* control or NTC samples were scored as containing positive droplets, the amplitude of the “positive” droplets is far less than the 20,000 average observed for wells containing *H*. *armigera* processed using the final optimized bulk extraction protocol (Figs 4 and 5). Post-processing of droplet data using the program “definetherain” greatly improved our ability to call positive droplets and eliminated ambiguity for intermediate droplets (“rain”). Results using fluorescence thresholds determined by “definetherain” were more repeatable than simply relying on QuantaSoft’s default threshold algorithm.

Data generation with ddPCR is fast, reproducible, and scalable. The rate limiting step for the experiments presented here is removing an individual leg from each moth. The workflow presented here could be improved by incorporating Bio-Rad’s plate-based AutoDG Automated Droplet Generator and a more rapid sampling method, possibly by grinding whole moths rather than removing individual legs. Using the current protocol, up to 1,000 moths can be processed in a single well, allowing for screening of up to 96,000 moths on a single plate, and providing a very efficient method for screening for *H*. *armigera* in the United States.

## Supporting information

S1 Table Raw data used to produce Fig 2. (ZIP)

S2 Table Raw data used to produce Fig 3. (ZIP)

S3 Table Raw data used to produce Fig 4. (ZIP)

S4 Table Raw data used to produce Fig 5. (ZIP)

S5 Table Raw data used to produce Fig 6. (ZIP)

## References

[1] Hardwick DF. The corn earworm complex. Memoirs of the Entomol Soc Can. 1965; 40: 1–247.

[2] Matthews M. Heliothine moths of Australia: a guide to pest bollworms and related noctuid groups Monographs on Australian Lepidoptera, Volume 7 CSIRO Publishing, Collingwood, Victoria, Australia; 1999.

[3] Cunningham JP, Zalucki MP. Understanding heliothine (Lepidoptera: Heliothinae) pests: What is a host plant? J Econ Entom. 2014; 107: 881–896. 25026644 10.1603/ec14036

[4] Perera OP, Walsh TK, Luttrell RG. Complete mitochondrial genome of *Helicoverpa zea* (Lepidoptera: Noctuidae) and expression profiles of mitochondrial-encoded genes in early and late embryos. J Insect Sci. 2016; 16: 40 doi: 10.1093/jisesa/iew023 27126963 10.1093/jisesa/iew023 PMC4864584

[5] Czepak C, Albernaz KC, Vivan LM, Guimarães HO, Carvalhais T. Research note. First reported occurrence of *Helicoverpa armigera* (Hübner) (Lepidoptera: Noctuidae) in Brazil. Pesq Agropec Trop. 2013; 43: 110–113.

[6] Tay WT, Soria MF, Walsh T, Thomazoni D, Silvie P, Behere GT, et al A brave new world for an Old World pest: *Helicoverpa armigera* (Lepidoptera: Noctuidae) in Brazil. PLoS ONE. 2013; 8(11): e80134 doi: 10.1371/journal.pone.0080134 24260345 10.1371/journal.pone.0080134 PMC3832445

[7] Gilligan TM, Tembrock LR, Farris RE, Barr NB, van der Straten MJ, van de Vossenberg BTLH, et al A multiplex real-time PCR assay to diagnose and separate *Helicoverpa armigera* and *H*. *zea* (Lepidoptera: Noctuidae) in the New World. PLoS ONE. 2015; 10(11): e0142912 doi: 10.1371/journal.pone.0142912 26558366 10.1371/journal.pone.0142912 PMC4641610

[8] Sosa-Gomez DR, Specht A, Paula-Moraes SV, Lopes-Lima A, Yano SAC, Micheli A et al Timeline and geographical distribution of *Helicoverpa armigera* (Hubner) (Lepidoptera, Nocuidae: Hleiothinae) in Brazil. Rev Bras Entomol. 2016; 60: 101–104

[9] Hayden J, Brambila J. Pest alert: Helicoverpa armigera (Lepidoptera: Noctuidae), the Old World bollworm. Florida Department of Agriculture and Consumer Services. 2015 Jun 17. Available: http://www.freshfromflorida.com/Divisions-Offices/Plant-Industry/Plant-Industry-Publications/Pest-Alerts/Pest-Alert-The-Old-World-Bollworm.

[10] Kriticos DJ, Ota N, Hutchison WD, Beddow J, Walsh T, Tay WT, et al The potential distribution of invading *Helicoverpa armigera* in North America: Is it just a matter of time? PLoS ONE. 2015; 10(3): e0119618 doi: 10.1371/journal.pone.0119618 25786260 10.1371/journal.pone.0119618 PMC4364701

[11] Behere GT, Tay WT, Russell DA, Heckel DG, Appleton BR, Kranthi KR, et al Mitochondrial DNA analysis of field populations of *Helicoverpa armigera* (Lepidoptera: Noctuidae) and of its relationship to *H*. *zea*. BMC Evol Biol. 2007; 7: 117 doi: 10.1186/1471-2148-7-117 17629927 10.1186/1471-2148-7-117 PMC1934911

[12] Pogue MG. A new synonym of *Helicoverpa zea* (Boddie) and differentiation of adult males of *H*. *zea* and *H*. *armigera* (Hübner) (Lepidoptera: Noctuidae: Heliothinae). An Entomol Soc Am. 2004; 97: 1222–1226.

[13] Brambila J. Instructions for dissecting male genitalia of Helicoverpa (Lepidoptera: Noctuidae) to separate H. zea from H. armigera. 2009. USDA-APHIS-PPQ http://www.aphis.usda.gov/plant_health/plant_pest_info/owb/downloads/owb-screeningaids2.pdf [unpublished instructional document].

[14] Hartstack AW, Witz JA, Buck DR. Moth traps for the tobacco budworm. J. Econ. Entomol. 1979; 72: 519–522.

[15] Guerrero S, Brambila J, Meagher RL. Efficacies of four pheromone-baited traps in capturing male *Helicoverpa* (Lepidoptera: Noctuidae) moths in Northern Florida. Fla Entomol. 2014; 97: 1671–1678.

[16] USDA (U.S. Department of Agriculture). New pest response guidelines: Helicoverpa armigera (Hübner) (Old World bollworm). 2014. Washington, D.C. http://www.aphis.usda.gov/import_export/plants/manuals/online_manuals.shtml.

[17] Ming Q-L, Wang C-Z. Genetic differentiation of *Helicoverpa armigera* (Hübner) and *H*. *assulta* (Guenée) (Lepidoptera: Noctuidae) based on AFLP markers. Insect Sci. 2006; 13: 437–444.

[18] Kranthi S, Kranthi KR, Bharose AA, Syed SN. A PCR-RFLP tool for differentiating *Helicoverpa armigera* and *H*. *assulta* (Lepidoptera: Noctuidae). Curr Sci. 2005; 89: 1322–1323.

[19] Li Q-Q, Li D-Y, Ye H, Liu X-F, Shi W, Cao N, et al Using COI gene sequence to barcode two morphologically alike species: the cotton bollworm and the oriental tobacco budworm (Lepidoptera: Noctuidae). Mol Biol Rep. 2011; 38: 5107–5113. doi: 10.1007/s11033-010-0658-1 21181271 10.1007/s11033-010-0658-1

[20] Leite NA, Alves-Pereira A, Corrêa AS, Zucchi MI, Omoto C. Demographics and genetic variability of the New World bollworm (*Helicoverpa zea*) and the Old World bollworm (*Helicoverpa armigera*) in Brazil. PLoS One. 2014; 9(11): e113286 doi: 10.1371/journal.pone.0113286 25409452 10.1371/journal.pone.0113286 PMC4237417

[21] Mastrangelo T, Paulo DF, Bergamo LW, Morais EGF, Silva M, Bezerra-Silva G, et al Detection and genetic diversity of a heliothine invader (Lepidoptera: Noctuidae) from North and Northeast of Brazil. J Econ Entomol. 2014; 107: 970–980. 25026655 10.1603/ec13403

[22] Nagoshi RN, Gilligan TM, Brambila J. Combining Tpi and CO1 genetic markers to discriminate invasive *Helicoverpa armigera* from local *Helicoverpa zea* (Lepidoptera: Noctuidae) populations in the southeastern United States. J Econ Entom. 2016; 109: 2115–2124. doi: 10.1093/jee/tow177 27551148 10.1093/jee/tow177

[23] Perera OP, Allen KC, Jain D, Purcell M, Little NS, Luttrell RG. Rapid identification of *Helicoverpa armigera* and *Helicoverpa zea* (Lepidoptera: Noctuidae) using ribosomal RNA Internal Transcribed Spacer 1. J Insect Sci. 2015; 15:155 doi: 10.1093/jisesa/iev137 26516166 10.1093/jisesa/iev137 PMC4625950

[24] Hindson BJ, Ness KD, Masquelier DA, Belgrader P, Heredia NJ, Makarewicz AJ, et al High-throughput droplet digital PCR system for absolute quantitation of DNA copy number. Anal Chem. 2011; 83: 8604–8610. doi: 10.1021/ac202028g 22035192 10.1021/ac202028g PMC3216358

[25] McDermott GP, Do D, Litterst CM, Maar D, Hindson CM, Steenblock ER, et al Multiplexed target detection using DNA-binding dye chemistry in droplet digital PCR. Anal Chem. 2013; 85, 11619–11627. doi: 10.1021/ac403061n 24180464 10.1021/ac403061n

[26] Bio-Rad [#1]. Droplet Digital PCR: QX200 ddPCR EvaGreen Supermix. Bulletin 6473, Rev A [undated]; 2 pp. Available: http://www.bio-rad.com/webroot/web/pdf/lsr/literature/Bulletin_6473.pdf.

[27] Nathan LM, Simmons M, Wegleitner BJ, Jerde CL, & Mahon AR. Quantifying environmental DNA signals for aquatic invasive species across multiple detection platforms. Environ Sci Technol. 2014; 48: 12800–12806. doi: 10.1021/es5034052 25299381 10.1021/es5034052

[28] Cao Y, Raith MR, Griffith JF. Droplet digital PCR for simultaneous quantification of general human-associated fecal indicators for water quality assessment. Water Res. 2015; 70: 337–349. doi: 10.1016/j.watres.2014.12.008 25543243 10.1016/j.watres.2014.12.008

[29] Floren C, Wiedemann I, Brenig B, Schutz E, Beck J. Species identification and quantification in meat and meat products using droplet digital PCR (ddPCR). Food Chem. 2015; 173: 1054–1058. doi: 10.1016/j.foodchem.2014.10.138 25466124 10.1016/j.foodchem.2014.10.138

[30] Gerdes L, Iwobi A, Busch U, Pecoraro S. Optimization of digital droplet polymerase chain reaction for quantification of genetically modified organisms. Biomol Det Quant. 2016; 7: 9–20. 10.1016/j.bdq.2015.12.003 PMC4827695 27077048

[31] Gloor GB, Preston CR, Johnson-Schlitz DM, Nassif NA, Phillis RW, Benz WK, et al Type I repressors of P element mobility. Genetics. 1993; 135: 81–95. 8224830 10.1093/genetics/135.1.81 PMC1205629

[32] Jones M, Williams J, Gärtner K, Phillips R, Hurst J, Frater J. Low copy target detection by Droplet Digital PCR through application of a novel open access bioinformatic pipeline, ‘definetherain.’ J Virol Methods. 2014; 202: 46–53. doi: 10.1016/j.jviromet.2014.02.020 24598230 10.1016/j.jviromet.2014.02.020 PMC4003534

[33] Bio-Rad [#2]. Droplet Digital PCR: Applications guide. Bulletin 6407, Rev A [undated]; 111 pp. Available: http://www.bio-rad.com/webroot/web/pdf/lsr/literature/Bulletin_6407.pdf.

[34] Armbruster DA, Pry T. Limit of blank, limit of detection and limit of quantitation. Clin Biochem Rev. 2008; 29: S49–S52. 18852857 PMC2556583

[35] Pinheiro LB, Coleman VA, Hindson CM, Herrmann J, Hindson BJ, Bhat S, et al Evaluation of a droplet digital polymerase chain reaction format for DNA copy number quantification. Anal Chem. 2012; 84: 1003–1011. doi: 10.1021/ac202578x 22122760 10.1021/ac202578x PMC3260738

